# Cell-penetrating peptides TAT and 8R functionalize P22 virus-like particles to enhance tissue distribution and retention *in vivo*

**DOI:** 10.3389/fvets.2024.1460973

**Published:** 2024-09-03

**Authors:** Shibo Su, Xuegang Shen, Xinqi Shi, Xin Li, Jin Chen, Wei Yang, Mingxia Sun, Yan-Dong Tang, Haiwei Wang, Shujie Wang, Xuehui Cai, Yu Lu, Tongqing An, Yongbo Yang, Fandan Meng

**Affiliations:** ^1^State Key Laboratory for Animal Disease Control and Prevention, Harbin Veterinary Research Institute of Chinese Academy of Agricultural Sciences, Harbin, China; ^2^Institute of Veterinary Immunology and Engineering, Jiangsu Academy of Agricultural Sciences, Nanjing, China; ^3^GuoTai (Taizhou) Center of Technology Innovation for Veterinary Biologicals, Taizhou, China; ^4^College of Veterinary Medicine, Northeast Agricultural University, Harbin, China; ^5^Heilongjiang Research Center for Veterinary Biopharmaceutical Technology, Harbin Veterinary Research Institute, Chinese Academy of Agricultural Sciences, Harbin, China; ^6^Heilongjiang Provincial Key Laboratory of Veterinary Immunology, Harbin Veterinary Research Institute, Chinese Academy of Agricultural Sciences, Harbin, China

**Keywords:** biological nanoparticle, P22 VLPs, TAT, 8R, cellular uptake, tissue distribution

## Abstract

Virus-like particles (VLPs) are used as nanocontainers for targeted drug, protein, and vaccine delivery. The phage P22 VLP is an ideal macromolecule delivery vehicle, as it has a large exterior surface area, which facilitates multivalent genetic and chemical modifications for cell recognition and penetration. Arginine-rich cell-penetrating peptides (CPPs) can increase cargo transport efficiency *in vivo*. However, studies on the tissue distribution and retention of P22 VLPs mediated by TAT and 8R are lacking. This study aimed to analyze the TAT and 8R effects on the P22 VLPs transport efficiency and tissue distribution both *in vitro* and *in vivo*. We used a prokaryotic system to prepare P22 VLP self-assembled particles and expressed TAT-or 8R-conjugated mCherry on the VLP capsid protein as model cargoes and revealed that the level of P22 VLP-mCherry penetrating the cell membrane was low. However, both TAT and 8R significantly promoted the cellular uptake efficiency of P22 VLPs *in vitro*, as well as enhanced the tissue accumulation and retention of P22 VLPs *in vivo*. At 24 h postinjection, TAT enhanced the tissue distribution and retention in the lung, whereas 8R could be better accumulation in brain. Thus, TAT was superior in terms of cellular uptake and tissue accumulation in the P22 VLPs delivery system. Understanding CPP biocompatibility and tissue retention will expand their potential applications in macromolecular cargo delivery.

## 1 Introduction

The rapid development of nanotechnology has attracted extensive attention, especially in the field of medical sciences, including in preventing infectious diseases, combating antibiotic-resistant bacteria, treating cancer, and enhancing drug delivery efficiency (1–4). Compared with other chemically synthesized nanomaterials, biologically derived nanoparticles, such as ferritins (5, 6) and virus-like particles (VLPs) (7–10), have obvious advantages, such as homogeneity and ease of genetic and chemical modification. The particle size is approximately 10–200 nm, which is beneficial for tissue permeability and retention, tissue distribution and bioaccumulation *in vivo* (11, 12). However, the small size of VLPs also affects the cargo carrying capacity. VLPs derived from phage P22 have an icosahedral structure of approximately 60 nm with a large surface area and internal space (13–15). It is self-assembled from approximately 420 copies of coat protein (CP) and 100–300 copies of scaffold protein (SP) (16). Both CP and SP are tolerant of genetic fusion without disrupting the assembly process of VLPs. To meet the special requirements of various applications, surface modification and internal encapsulation are the primary strategies for VLPs modification.

Chemical cross-linking or genetic modification of the capsid structure may decorate VLPs with diverse structures (17). Internal encapsulation is achieved through N-terminal fusion with the scaffold protein SP. O’Neil et al. (18) successfully encapsulated functional proteins within VLPs and achieved controlled release of cargo from the capsid container. To date, cargo sizes ranging from 20 kDa to 180 kDa have been effectively encapsulated by P22 VLPs. In addition, Parent et al. (19) reported that the phage L trimer decorator (Dec) protein has high affinity for P22 CP and was used to increase the surface area of P22 VLPs by adding cargoes to either end of Dec, which may offer a total of 80 potential binding sites capable of binding 240 decoration protein monomers (20). Additionally, fusion expression with the C-terminus of the CP protein is another approach for P22 VLPs surface modification. Li et al. (14) reported that P22 VLPs fused with the T epitope of ovalbumin at the C-terminus of CP can strongly activate a T epitope-specific CTL response and increase antitumor effects.

Although P22 VLPs have good tissue distribution and retention *in vivo*, their ability to penetrate cell membrane barriers needs to be enhanced, especially for tissues with a continuous endothelium, which limits nanoparticle delivery efficiency. Some studies have utilized chemical coupling methods to modify receptors or ligands such as estradiol, EFGR, and HER2 on the surface of P22 VLPs to improve their cellular uptake efficiency (21). However, chemical coupling has disadvantages such as potential toxicity, complex operating procedures, variability between batches, and high production costs. Cell-penetrating peptides (CPPs), also known as protein transduction domains, exhibit broad cell applicability. CPPs are short peptides capable of transporting proteins, peptides, and other biological macromolecules across mammalian cell membranes, allowing therapeutic substances to directly enter cells and maintain their biological activity (22, 23). CPPs can penetrate various cell types and even cross the blood-brain barrier (BBB) *in vivo* (24). Although the CPP membrane penetration mechanism has been debated, it is generally categorized into direct membrane penetration (energy-independent pathway) and energy-dependent endocytosis. The specific membrane penetration process is influenced by the physical and chemical properties of the CPP itself, as well as the cargo being carried (25). Anand et al. (26) conjugated TAT, a natural CPP originally discovered in HIV, to the capsid of P22 VLPs and demonstrated that the engineered VLPs not only efficiently penetrated the cell membrane but also successfully overcame the BBB. A previous study suggested that the endocytic mechanism and intracellular fate of CPP-mediated cellular uptake strongly depend on the attached cargo (27). The effects of different CPPs on the cellular uptake efficiency and tissue distribution *in vivo* of the cargo remain unclear.

Although both TAT and 8R are polyarginine transmembrane peptides, the influences of the presence of charged and hydrophobic residues of peptide are not only the uptake capacity but also cytotoxicity. So far, the differences of TAT and 8R in regulating cargo uptake remain unclear. Therefore, in this study we explored the effects of TAT and 8R on mediating the transport efficiency and tissue distribution of P22 VLPs both *in vitro* and *in vivo*. We used a prokaryotic system to prepare phage P22 VLP self-assembled particles and expressed TAT-or 8R-conjugated mCherry on the capsid protein of VLPs as model cargoes. Our results demonstrated that TAT and 8R could significantly increase the cellular uptake efficiency and accumulation of P22 VLPs *in vivo*. Additionally, TAT notably enhances the tissue distribution and retention in the lung, whereas 8R exhibited greater accumulation in brain. These findings indicate that, through the modification of CPPs, P22 VLPs have the potential to serve as an effective delivery system for macromolecular drugs and vaccines, providing new perspectives and strategies for the application of bionanotechnology in the medical field.

## 2 Materials and methods

### 2.1 Ethics statement

All the animal experiments were conducted in accordance with the Guide for the Care and Use of Laboratory Animals of the Ministry of Science and Technology of the People’s Republic of China, and the animal experiments (231207-03-GR) were performed under the supervision of the Committee on the Ethics of Animal Experiments of the Harbin Veterinary Research Institute (HVRI) of the Chinese Academy of Agricultural Sciences (CAAS) and the Animal Ethics Committee of Heilongjiang Province, China.

### 2.2 Cells, proteins and plasmids

HEK293T (human embryonic kidney) cells and Marc-145 (African green monkey kidney) cells were maintained in Dulbecco’s modified Eagle’s medium (DMEM; Gibco, United States) supplemented with 10% fetal bovine serum (FBS; Gibco, United States). The pET-28a-mCherry-6 × His plasmid was synthesized and transformed into ROSETTA competent cells, and expression was induced by 0.1 mM IPTG for 18 h at 16°C. The soluble mCherry protein was purified via nickel affinity chromatography. Four nanoparticles, P22 VLP, P22 VLP-mCherry, P22 VLP-mCherry-TAT, and P22 VLP-mCherry-8R, were prepared via a prokaryotic expression system. The P22 CP and SP (aa 141–303) genes were synthesized as described in a previous study (28, 29) and amplified via homologous recombination primers. SP was cloned downstream of the T7 promoter in the pET-28a vector, whereas CP was inserted after the SP stop codon with an added ATG at its 5′ end for expression initiation. The final construct obtained was pET-28a-P22 VLP. Furthermore, the mCherry, mCherry-TAT, and mCherry-8R sequences were amplified with the corresponding primers and inserted into the C-terminus of the CP gene in pET-28a-P22 VLP to construct pET-28a-P22 VLP-mCherry-TAT and pET-28a-P22 VLP-mCherry-8R. All primers were synthesized by Jilin Kumei Biotechnology Co., Ltd., and the sequences are shown in Supplementary Table S1.

### 2.3 Expression and purification of P22 VLPs

The four P22 VLP vectors were transformed into Rosetta (DE3) competent cells, which were subsequently cultured at 37°C overnight. Single clones were selected for expansion, and the VLPs were induced at 16°C for 20 h with 0.1 mM IPTG for expression. Then the cells were harvested by centrifugation at 3,700 × g for 20 min. The cell pellets were suspended in PBS (pH 7.0) and stored at −80°C until further use. The cells were lysed by sonication, and the supernatants were collected for SDS-PAGE after centrifugation. The molecular weights of each component protein in VLPs were predicted by SnapGene 6.02 software. The filtered supernatants were then transferred to 40 mL polypropylene centrifuge tubes and centrifuged at 120,000 × g for 2 h with ultracentrifugation (Beckman Coulter Optima XPN-100) at 4°C. For purification, the concentrated proteins were carefully added on top of four sucrose concentration gradients (25–55%) prepared in 40 mL centrifuge tubes and centrifuged at 120,000 × g for 3 h at 4°C. The VLP band was carefully collected and dissolved in PBS, followed by centrifugation to remove any remaining sucrose. The purified VLPs were further confirmed via SDS-PAGE, and then small aliquots were prepared and stored at −20°C (21). The VLP concentrations were determined using a BCA protein concentration kit (Thermo Fisher Scientific, United States). The sizes of the VLP particles were analyzed by dynamic light scattering (DLS) via a nanoparticle size analyzer (Malven Nano2S90, United Kingdom) and Zwtasizer software as previous study (14).

### 2.4 Electron microscope observation of P22 VLPs morphology

The morphological characteristics of the virus-like particles were detected via transmission electron microscopy (TEM). The samples were applied to a copper grid and incubated at 25°C for 10 min. A 2.5% phosphotungstic acid solution was subsequently used for negative staining of the samples for 1 min. Excess stain was removed by blotting with filter paper, and the prepared samples were examined via transmission electron microscopy (H-7650, Hitachi, Tokyo, Japan). For immunoelectron microscopy, a mouse mCherry monoclonal antibody was used at a dilution of 1:200 and visualized with a Gold-labeled anti-mouse secondary antibody.

### 2.5 Live-cell confocal microscopy observation of P22 VLPs penetration

HEK293T cells and Marc-145 cells were seeded in confocal dishes at a density of 1 × 10^6^ and cultured overnight at 37°C and 5% CO_2_. The purified VLP-mCherry, VLP-mCherry-TAT and VLP-mCherry-8R were added to HEK293T and Marc-145 cells at a final concentration of 10 μg/mL for 1 or 12 h of incubation. The cells were subsequently incubated with CellMask^™^ Green Plasma Membrane Stain (Thermo Fisher Scientific, United States) and NucBlue^™^ Live Cell Stain ReadyProbes^™^ (Thermo Fisher Scientific, United States) at 37°C for 10 min. After washing 3 times with PBS, the culture medium was replaced with fresh medium. The fluorescence distribution of mCherry was observed via confocal laser scanning microscopy (LSM980-ZEISS). The fluorescence intensity of each group was calculated via ImageJ software, and the results are presented as the percentage of mCherry fluorescence relative to the nuclear fluorescence intensity.

### 2.6 Western blot analysis of mCherry delivery efficiency

HEK293T cells were incubated with different concentrations of P22 VLPs for 3 h, after which the cell lysate supernatant was collected, mixed with 5× loading buffer and separated by 12% SDS-PAGE. Following the transfer of proteins from the gel to a PVDF membrane (Millipore, United States), the membrane was blocked with 5% skim milk for 1 h at room temperature. The samples were then incubated with specific primary antibodies overnight at 4°C. After three washes with PBST, the membrane was incubated with the secondary antibody for 1 h at room temperature. Signal detection was carried out via a near-infrared fluorescence scanning imaging system (Odyssey CLX). The primary antibodies used were mouse anti-mCherry tag mAb (1:5,000 dilution; Sigma-Aldrich, United States), rabbit anti-His tag mAb (1:5,000 dilution) and mouse anti-β-actin tag mAb (1:10,000 dilution). The secondary antibodies, diluted 1:10,000 in PBS, were DyLight 800-labeled goat anti-mouse IgG (KPL, United States) or DyLight 800-labeled goat anti-rabbit IgG (KPL, United States).

### 2.7 Cell viability analysis

Cell viability was assessed with a cell counting kit-8 (CCK-8) (Dojindo, Japan) following the manufacturer’s instructions. HEK293T cells were initially seeded at a density of 2 × 10^4^ cells per well in 96-well plates and then incubated in a 5% CO_2_ humidified incubator at 37°C. Once a confluent monolayer was observed, the cells were washed with PBS and incubated with different concentrations of the three P22 VLPs in serum-free medium for 24 h. Subsequently, 10 μL of CCK-8 reagent was added to each well of a 96-well plate containing 100 μL of fresh medium and incubated for 2 h at 37°C. The absorbance was measured at 450 nm via a Multiscan Spectrum (PE, Enspire, United States), and the cell viability was calculated. The results are presented as the percentage of the optical density of P22 VLP-treated cells compared with that of the untreated control cells, and the untreated cells were considered 100% viable.

### 2.8 Live fluorescence imaging of mice

For the mouse experiments, twenty-five 6-week-old BALB/c mice weighing between 18 and 22 g were randomly divided into 5 groups and injected with purified VLP-mCherry, VLP-mCherry-TAT, VLP-mCherry-8R nanoparticles, mCherry protein or PBS by tail vein (10 mg/kg). Then the distribution of P22 VLPs was detected via live fluorescence imaging. The fluorescence intensities were monitored before tail vein injection (0 h) and at 1, 6, 12, 24 and 48 h after injection. Mice in a completely comatose state were placed on the detection platform of a small animal *in vivo* imaging system (BERTHOLD LB 983 NC100) in a uniform lying position on the left side, and signals were collected at an excitation wavelength of 500 nm and an emission wavelength of 600 nm. After *in vivo* imaging, the mice were sacrificed for organ collection, and the fluorescence intensity of the organs was detected with the same parameters. The relevant parameters were adjusted based on the mCherry fluorescence intensity of three purified VLPs displayed in the tube. The data were collected via IndiGO 2 software. The results are presented as the mean fluorescence intensity and standard error and were analyzed via GraphPad 8.0.

### 2.9 Statistical analysis

All experiments were performed with three independent replicates, and the error bars indicate the standard deviation (SD). Statistically significant differences were analyzed by two-way ANOVA with GraphPad Prism 8.0 software. A *p*-value <0.05 was considered to indicate statistical significance.

## 3 Results

### 3.1 Design and characterization of virus-like particles

**Figure 1:**
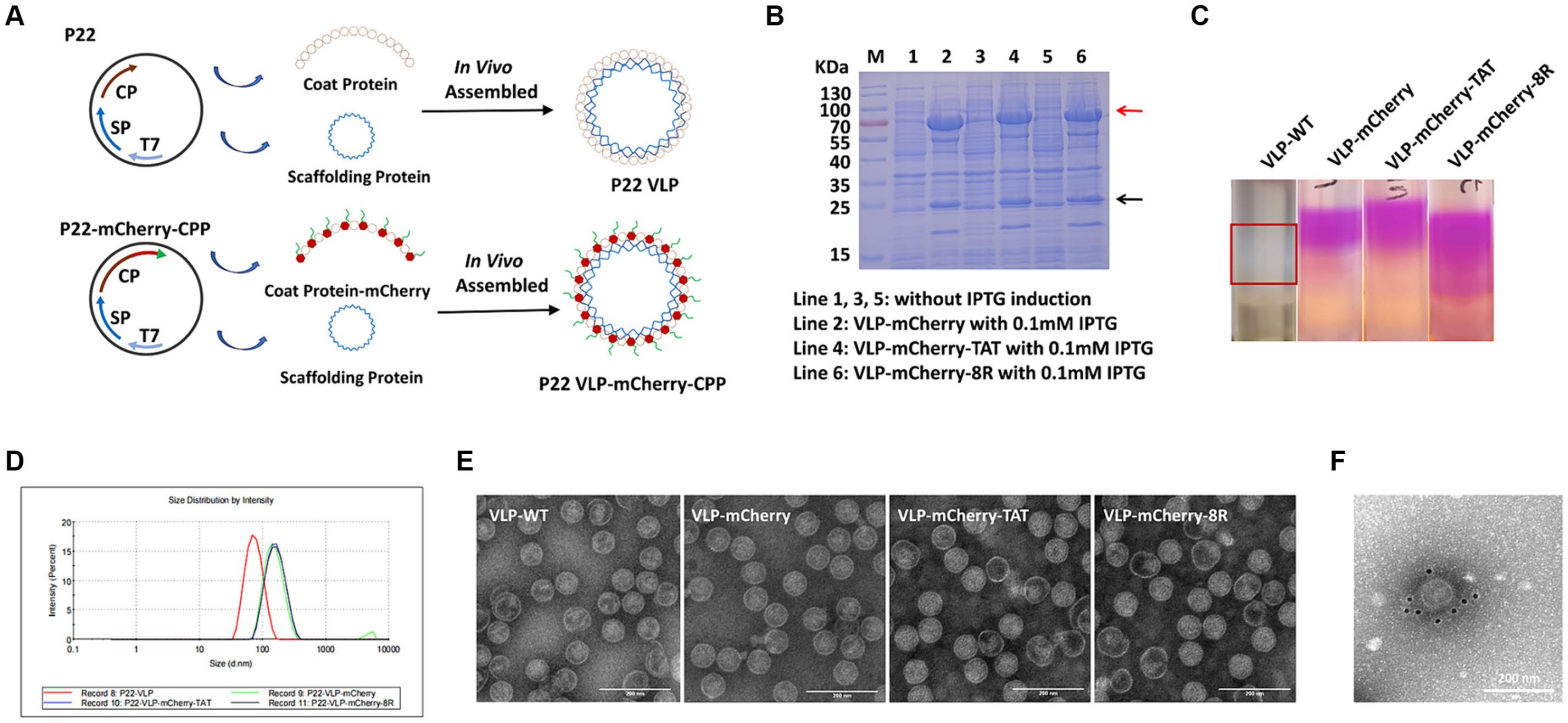
Assembly and purification of P22 VLPs. **(A)** Schematic illustration of the formation of naked-P22-VLPs and modified VLPs containing CPP-conjugated mCherry. **(B)** Characterization of the expression of VLP-mCherry, VLP-mCherry-TAT, and VLP-mCherry-8R by SDS-PAGE. M: protein marker; lines 1, 3, and 5: without IPTG induction; lines 2, 4, and 6: with 0.1 mM IPTG induction of the indicated P22 VLPs. **(C)** Purification of P22 VLPs via sucrose density gradient centrifugation. **(D)** Particle size analysis. **(E)** Transmission electron microscopy (TEM) imaging. Scale bar: 200 nm. **(F)** Immunoelectron microscopy imaging of VLP-mCherry. Scale bar: 200 nm.

P22 VLPs and mCherry were used as vectors and indicator proteins to analyze the efficiency of cell-penetrating peptide (CPP)-mediated nanoparticles carrying large proteins to penetrate the cell membrane. The vector pET-28a containing the T7 promoter was used for expression of the P22 VLP particles. Servid et al. (30) confirmed that the C-terminus of the CP protein is displayed on the surface of the particle, so we cloned the mCherry that fused with the CPP into the C-terminus of the coat protein (CP) and was connected by the linker GGGGS (Figure 1A). The VLPs that carried mCherry, mCherry-TAT or mCherry-8R were named VLP-mCherry, VLP-mCherry-TAT or VLP-mCherry-8R, respectively. VLP-WT was cloned as a negative control. VLPs were expressed in *E. coli*, and the supernatants were collected for SDS-PAGE and purification after the competent cells were lysed by sonication and centrifugation. The SDS-PAGE analysis of VLP-WT illustrated the size of SP protein was 22.5 kDa and the CP monomer was approximately 50 kDa, which were expressed in the presence of 0.1 mM IPTG (Supplementary Figure S1). While, the molecular weight of the SP of VLP-mCherry, VLP-mCherry-TAT and VLP-mCherry-8R was 22.5 kDa, and the bands for CP-mCherry, CP-mCherry-TAT and CP-mCherry-8R had the correct molecular weights of approximately 75 kDa (Figure 1B). In addition, the band of CP monomer can be detected in VLP-mCherry, VLP-mCherry-TAT or VLP-mCherry-8R (Figure 1B). Western blot analysis was further performed using mCherry and His antibodies, respectively. The results revealed that the mCherry antibody labeled the mCherry-conjugated CP was approximately 75 kDa, and His antibody labeled the SP protein (22.5 kDa) and the CP monomer (50 kDa) (Supplementary Figure S2). The VLPs were purified via sucrose density gradient centrifugation, as shown in Figure 1C. mCherry expressed by VLP-mCherry, VLP-mCherry-TAT and VLP-mCherry-8R formed a pink layer after purification. Only a white layer was observed in VLP-WT. Pink or white fractions were carefully collected from the gradient, and the purified VLPs were analyzed by a nanoparticle size analyzer. The nanoparticles expressing mCherry were approximately 140 nm in diameter, whereas the particle size of VLP-WT was approximately 70 nm (Figure 1D). These results indicated that surface expression of mCherry increased the nanoparticle size. In addition, TEM imaging revealed that the three types of VLPs expressing mCherry with or without CPP self-assembled into nanospheres and were not notably different from VLP-WT (Figure 1E). These results confirmed that the expression of mCherry and CPP did not interfere with P22 VLPs self-assembly. To prove the successful localization of mCherry on the surface of P22 VLPs, immunoelectron microscopy was conducted. Briefly, purified VLP-mCherry labelled by primary antibody against mCherry, and mCherry was visualized via a secondary antibody coupled to a gold particle. The gold particles were attached to the surface of VLP-mCherry (Figure 1F), which indicates that mCherry was expressed on the surface of the P22 VLP nanoparticles.

### 3.2 TAT and 8R mediate P22 VLPs transport across the plasma membrane

**Figure 2:**
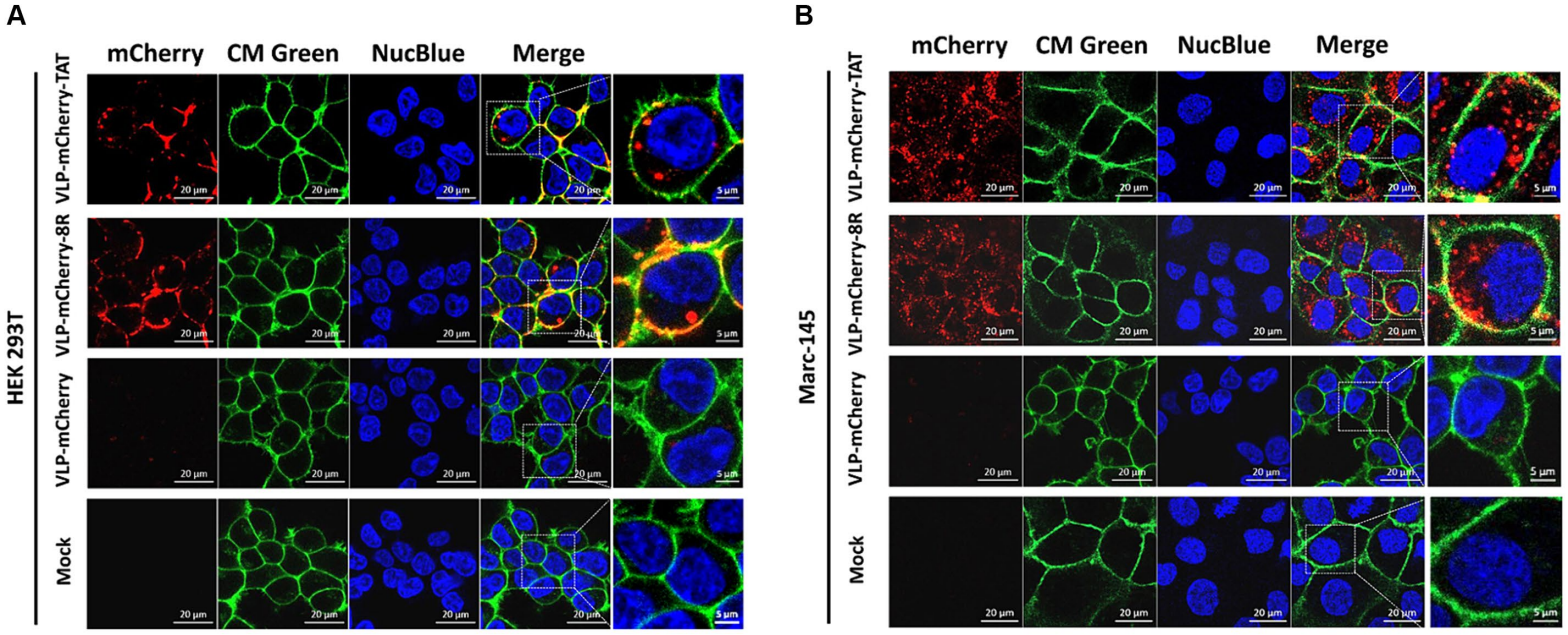
TAT and 8R mediate P22 VLPs transport across the plasma membrane in HEK293T and Marc-145 cells. VLP-mCherry, VLP-mCherry-TAT, and VLP-mCherry-8R were incubated with HEK293T cells for 1 h **(A)** or with Marc-145 cells for 12 h **(B)**. The cell nuclei were stained with NucBlue^™^ Live Cell Stain ReadyProbes^™^ (blue), and the cell membrane was visualized with CellMask^™^ Green Plasma Membrane Stain (green). The cellular uptake was monitored via live confocal microscopy. Scale bar: 20 μm.

To deliver cargo into cells, nanoparticles must be able to traverse the plasma membrane, and CPPs have been used to facilitate the translocation of cargo across the plasma membrane via endocytosis (31–35). To avoid artifactual redistribution throughout the cell during the cell fixation process (36), the effects of TAT-and 8R-mediated P22 VLPs uptake were monitored via live confocal microscopy. Briefly, HEK293T cells were incubated with 10 μg/mL VLPs for 1 h, after which the cell membrane (in green) and nucleus (in blue) were labeled with fluorescent dye to visualize the outer cell membrane and nucleus. The uptake of VLPs by cells was subsequently monitored via live confocal microscopy. Although most of the VLP-mCherry-TAT and VLP-mCherry-8R nanoparticles attached to the cell membrane colocalized with the cell membrane at 1 h postincubation, both TAT and 8R mediated P22 VLPs uptake by HEK293T cells, and a few red punctate spots were observed in the cytoplasm (Figure 2A). However, compared with VLP-mCherry-TAT or VLP-mCherry-8R nanoparticles, VLP-mCherry rarely colocalized with the HEK293T cell membrane, and the intracellular red aggregation signal was very weak. Compared with the cell lines commonly used for transient transfection, African green monkey kidney-derived cells (Marc-145) presented low transfection efficiency (37). Therefore, we further analyzed the effects of TAT and 8R on the efficiency with which P22 VLPs (10 μg/mL) enter Marc-145 cells under prolonged incubation. The results showed that both TAT and 8R were able to efficiently mediate P22 VLPs internalization into Marc-145 cells at 12 h postincubation, and large amounts of red aggregates appeared in the cytoplasm, whereas only a few nanoparticles were observed on the cell surface (Figure 2B). However, even with increasing incubation time, VLP-mCherry was rarely observed on the cell membrane or in the cytoplasm. These results indicated that the association of TAT and 8R with the cell membrane is important for proper initiation of P22 VLPs internalization.

### 3.3 TAT is more efficient than 8R at initiating P22 VLPs entry into cells

**Figure 3:**
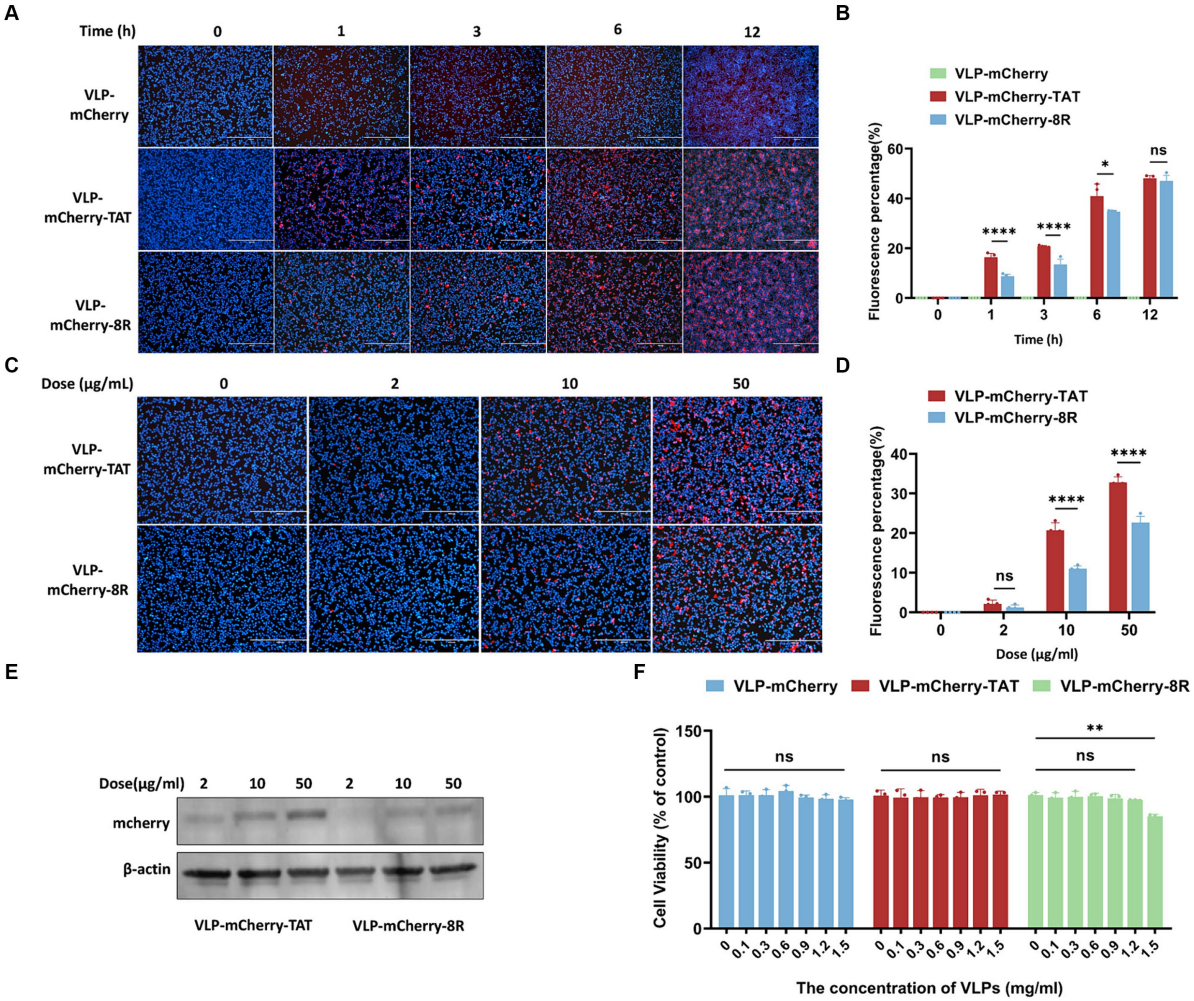
Efficiency of P22 VLPs uptake by HEK293T cells mediated by TAT and 8R. **(A)** HEK293T cells were incubated with 10 μg/mL of the indicated P22 VLPs for 0, 1, 3, 6 or 12 h. The cell nuclei were stained with NucBlue^™^ Live Cell Stain ReadyProbes^™^ (blue), and then, the HEK293T cells were imaged via confocal microscopy. Scale bar: 400 μm. **(B)** The fluorescence signals of **A** were calculated via ImageJ software. **(C)** HEK293T cells were incubated with various concentrations of the indicated P22 VLPs for 3 h. The cell nuclei were stained with NucBlue^™^ Live Cell Stain ReadyProbes^™^ (blue), and then, the HEK293T cells were imaged via confocal microscopy. Scale bar = 400 μm. **(D)** The fluorescence signals of C were calculated via ImageJ software, and **(E)** the mCherry expression level was analyzed via western blotting. **(F)** Cell viability analysis via the CCK8 assay. The data are presented as the means ± standard deviations (SDs) from three independent experiments. *p*-values were calculated via ANOVA as *p <* 0.05 (*), *p <* 0.01 (**), *p <* 0.001 (***), *p <* 0.0001 (****), and ns, not significant.

Although TAT and 8R have shown great potential for delivering macromolecular therapeutics into cells in cell culture and in animal disease models *in vivo*, the difference in the efficiency of TAT and 8R in mediating P22 VLPs entry into cells is unclear. To evaluate the efficiency of VLP-mCherry-TAT and VLP-mCherry-8R entry into cells, HEK293T cells were exposed to VLPs at 10 μg/mL, and live cells were observed by time-lapse confocal imaging, which revealed that the VLPs dispersed with the cells. Therefore, VLPs can be visualized continuously as they are adsorbed and internalized. As shown in Figure 3A, the delivery efficiency of P22 VLPs by TAT and 8R into HEK293T cells was time dependent, and the detectable punctate spots in VLP-mCherry-TAT-and VLP-mCherry-8R-treated cells increased from 1 to 12 h (Figure 3A). No visualized mCherry aggregates were detected in VLP-mCherry-or mock-treated cells. The percentage of red fluorescence was subsequently calculated and is presented in Figure 3B. At 1, 3, and 6 h, the uptake ratio of P22 VLPs mediated by TAT was significantly greater than that mediated by 8R in HEK293T cells (*p <* 0.05). In particular, at the initial stage of VLPs incubation, the percentage of mCherry fluorescence emitted by VLP-mCherry-TAT was twice that emitted by VLP-mCherry-8R at 1 h (*p <* 0.001). After 12 h, TAT-and 8R-mediated P22 VLPs entry reached similar levels, and the percentage of fluorescence reached 45% (*p* > 0.05) (Figure 3B). In addition, the efficiency of P22 VLPs delivery into cells with different concentrations of VLP-mCherry-TAT and VLP-mCherry-8R was evaluated. The efficiency of P22 VLPs uptake by HEK293T cells enhanced with increasing VLPs concentration (Figure 3C). The percentage of mCherry fluorescence in VLP-mCherry-TAT-treated cells was significantly greater than that in cells treated with 10 μg/mL or 50 μg/mL VLP-mCherry-8R after 3 h of incubation (Figure 3C). The fluorescence data are reflected in Figure 3D. In addition, the above cell lysates were collected, and the mCherry delivery efficiency was detected by WB. The results indicated that TAT-conjugated P22 VLPs mediate higher levels of mCherry delivery than 8R VLPs (Figure 3E), which was consistent with the fluorescence results (Figure 3D). A previous study suggested that the peptide composition, in terms of charged residues and hydrophobic residues, influences not only the uptake capability but also the cellular toxicity (38). The VLPs effect on the proliferation of HEK293T cells was detected, and the results of the cell viability analysis revealed that the concentration of VLP-mCherry-8R at 1.5 mg/mL significantly interfered with HEK293T cell proliferation (*p <* 0.01), whereas the effects were not influenced by VLP-mCherry-TAT or VLP-mCherry at the same concentration (Figure 3F). These results indicated that a high concentration of TAT-conjugated P22 VLPs has a weaker cytotoxic effect on host cells than does 8R.

### 3.4 TAT promotes efficient delivery of the P22 VLP-loaded cargo *in vivo*

**Figure 4:**
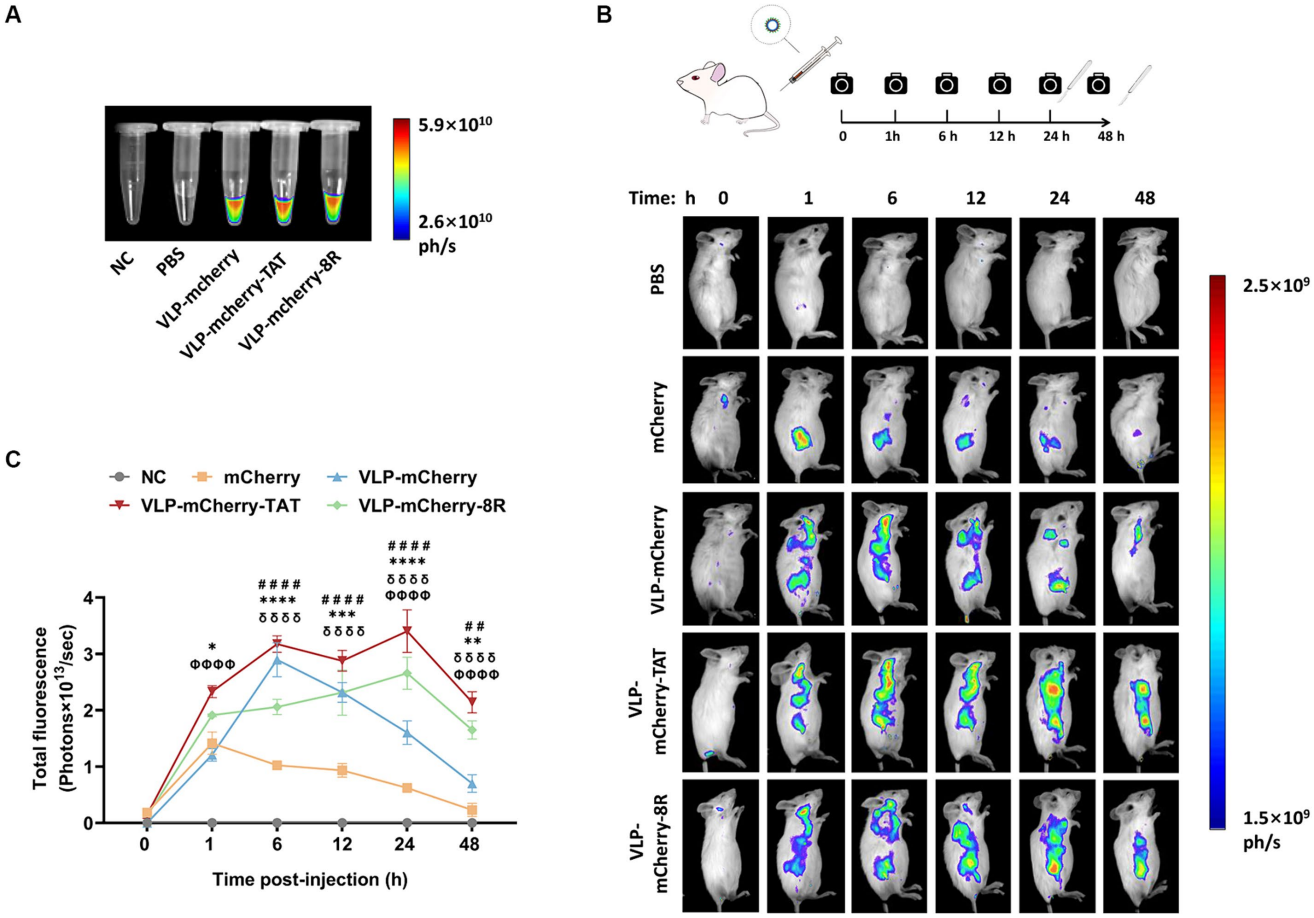
Delivery of CPP-conjugated P22 VLPs *in vivo*. **(A)** mCherry fluorescence in the EP tube was detected via an *in vivo* imaging system at appropriate wavelengths (*λ*ex =500 nm, *λ*em =600 nm), with empty tubes and PBS blank control groups. **(B)** Schematic diagram of the design of the *in vivo* imaging experiment and the distribution of mCherry fluorescence in mice at different time points after intravenous injection of mCherry, VLP-mCherry, VLP-mCherry-TAT or VLP-mCherry-8R. PBS was used as a blank control. All the images were scaled to the same minimum and maximum values. **(C)** Statistical analysis of the total fluorescence intensity of the *in vivo* imaging experiment. The data are shown as the means ± SDs. The asterisk (*) indicates a significant difference in total fluorescence between VLP-mCherry-TAT (red) and VLP-mCherry-8R (green) (* *p* < 0.05; ** *p* < 0.01; *** *p* < 0.001; **** *p* < 0.0001). Pound (#) indicates a significant difference between VLP-mCherry (blue) and mCherry (yellow) (## *p* < 0.01; #### *p* < 0.0001). Phi (φ) indicates a significant difference between VLP-mCherry-TAT (red) or VLP-mCherry-8R (green) and mCherry (yellow) (φφφφ *p* < 0.0001). Delta (δ) indicates a significant difference between VLP-mCherry-TAT (red) or VLP-mCherry-8R (green) and VLP-mCherry (blue) (δδδδ *p* < 0.0001).

*In vivo* imaging is an important optical method that uses luminescent materials to observe the biological distribution of target cargo within living tissue (39–41). Before conducting the *in vivo* imaging experiment, the fluorescence signal of the purified P22 VLPs was detected. The results revealed that high-intensity fluorescence signals were detected in the VLPs carrying mCherry, whereas no signal was detected in the blank tube (NC) or PBS (Figure 4A). To evaluate the tissue permeability and retention of CPP-conjugated VLPs delivering the mCherry protein *in vivo*, BALB/c mice were injected with PBS, mCherry, VLP-mCherry, VLP-mCherry-TAT, or VLP-mCherry-8R via the tail vein at a dose of 10 mg/kg, and *in vivo* imaging was conducted at the indicated time points (Figure 4B). The mice were monitored for physical activity and appearance following injection to assess any potential negative effects during whole experiment period. The appearance and physical activity of the mice in all groups remained unchanged, suggesting that P22 VLPs did not cause any negative effects. The fluorescence signal was detected in the bodies of the mice in all the mCherry protein-containing groups, indicating successful deep-tissue imaging. Except for the negative control, the fluorescence intensity in each group generally initially increased but then decreased (Figure 4B). The fluorescence distribution of mCherry delivered by P22 VLPs was wider, and the fluorescence intensity was stronger than that of naked mCherry (Figure 4B). These results indicate that P22 VLPs facilitate target cargo tissue delivery efficiency and biological distribution. Statistical analysis of the fluorescence values revealed that the fluorescence value of naked mCherry peaked at 1 h postinjection and was significantly lower than that of the other groups. Notably, the fluorescence intensity of VLP-mCherry dramatically decreased beginning at 6 h postinjection; however, the fluorescence of VLP-mCherry-CPP continuously increased until 24 h postinjection (Figure 4C). In addition, VLP-mCherry-CPP showed significantly stronger fluorescence intensity than VLP-mCherry or naked mCherry did at 48 h postinjection, highlighting the role of CPP-conjugated P22 VLPs in enhancing mCherry tissue retention (*p <* 0.0001). Furthermore, the fluorescence of VLP-mCherry-TAT-injected mice was the highest during the entire experimental period and was significantly greater than that of VLP-mCherry-8R-injected mice (*p <* 0.05), indicating that TAT-conjugated P22 VLPs have greater tissue delivery efficiency (Figure 4B).

### 3.5 CPPs facilitate P22 VLPs delivery of targeted cargo to the lungs and brain

**Figure 5:**
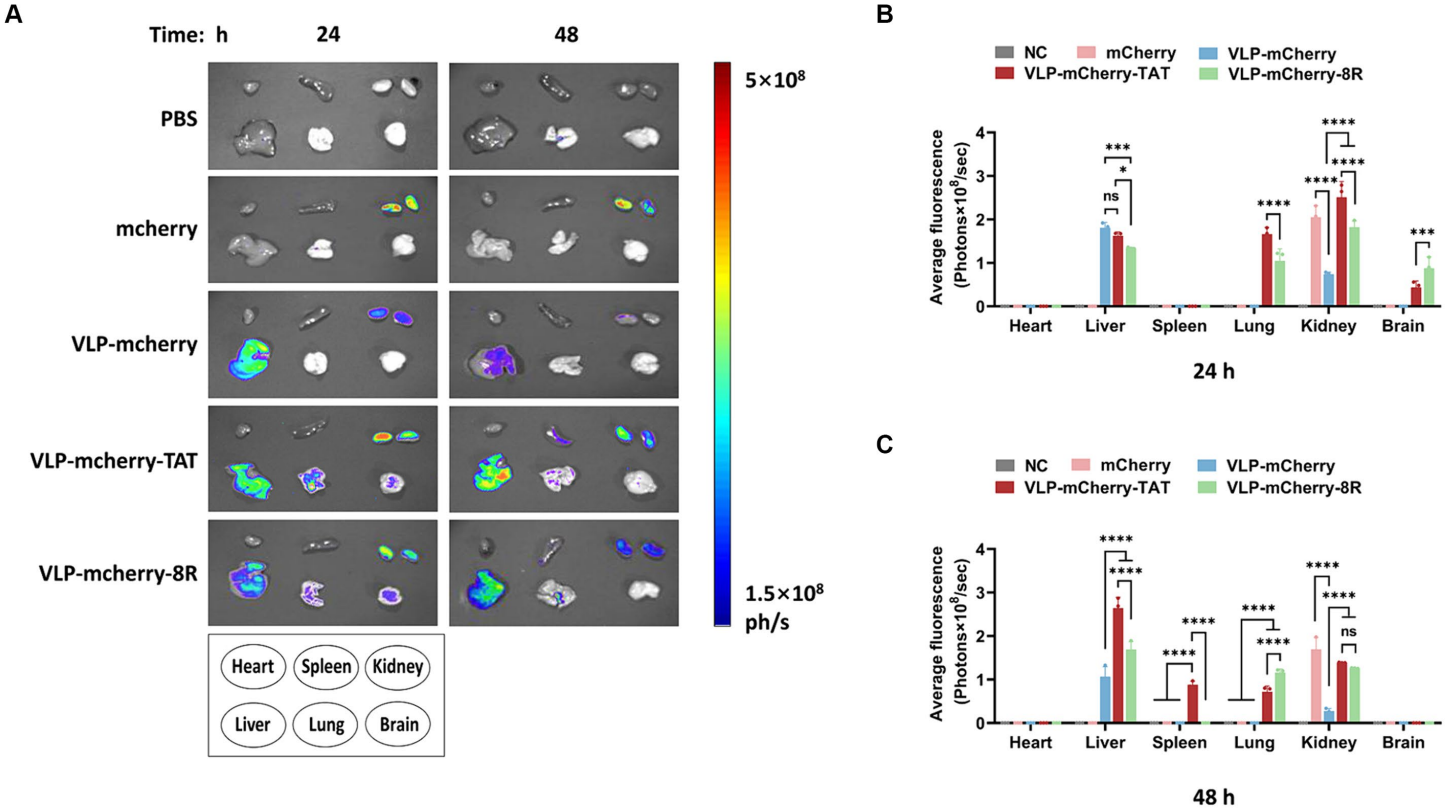
Distribution of P22-VLPs in various organs of the mice. **(A)** Various organs were collected at 24 h and 48 h after injection, and *ex vivo* imaging of the heart, liver, spleen, lung, kidney and brain was conducted via an *in vivo* imaging system. The average fluorescence value of each organ was statistically analyzed at 24 h **(B)** and 48 h **(C)** after injection. The data are presented as the means ± SDs. *p*-values were calculated via two-way ANOVA as *p <* 0.05 (*), *p <* 0.01 (**), *p <* 0.001 (***), *p <* 0.0001 (****), and ns, not significant.

One of the most important determinants of nanoparticle efficacy is their biodistribution profile and exposure at the site of action (42). To reveal the VLPs distribution in the mouse organs, various organs were collected for *ex vivo* tissue imaging. We detected distinct distribution patterns of mCherry when it was delivered via different vehicles. At 24 h postinjection, VLP-mCherry and naked mCherry were distributed in the liver and/or kidney (Figure 5A). While the CPP-conjugated VLPs had the widest tissue distribution, strong fluorescence signals were also detected in the lung and brain. Although both transmembrane peptides displayed varying pulmonary delivery efficiencies for P22 VLPs, the degree of tissue retention in the lungs was maintained for up to 48 h postinjection (Figure 5A). Statistical analysis of the fluorescence values revealed that only CPP-conjugated VLPs produced fluorescence signals in the lung and brain and that TAT induced significantly stronger signals in the lungs than did 8R (*p <* 0.0001), whereas 8R produced greater signals in the brain (*p <* 0.001) (Figure 5B). In addition, the signals of naked mCherry and CPP-conjugated VLPs in the kidney were significantly stronger than those of VLP-mCherry (Figure 5B). Furthermore, the CPP-conjugated VLPs presented increased fluorescence intensity in the liver at 48 h postinjection, and the fluorescence value of VLP-mCherry-TAT was significantly greater than that of VLP-mCherry-8R and VLP-mCherry (*p <* 0.0001). Interestingly, only VLP-mCherry-TAT presented fluorescence in the spleen (Figure 5C). These results suggest that the TAT and 8R peptides mediate broader tissue distribution and tissue retention of P22 VLPs, which facilitates tissue delivery of the target cargo *in vivo*.

## 4 Discussion

As archetypal biological nanoparticles, VLPs have the advantages of both viral and nonviral vectors. VLPs can utilize the advantages of viruses as well as cell uptake and transport mechanisms to deliver cargo into the cytoplasm (43–45). Compared with VLPs originating from pathogens, phage VLPs are a favorable choice for delivering biomacromolecule drugs or vaccines, as they are not affected by preexisting antibodies in mammals (46). Previous studies have demonstrated that P22 VLPs-WT themselves could effectively induce the activation of bone marrow-derived DCs, including upregulation of costimulatory molecules and cytokines expression, but it cannot effectively activate antigen cross-presentation on MHC-I compared to with antigen-loaded vaccine candidates, and P22 VLPs-WT induced low level of cellular immunity in mice (14, 47).

Our study demonstrated that employing P22 VLP vectors notably extended the duration of mCherry retention in mice compared to the naked protein, which is beneficial for the sustained efficacy of the delivered drugs or vaccines. Interestingly, although P22 VLP-mCherry produced high fluorescence signals in a mouse model and was distributed mainly in the liver and kidneys, we found that P22 VLP-mCherry exhibited very low uptake efficiency by both HEK293T cells and Marc-145 cells. P22 VLP-mCherry seems unable to bind successfully to the cell membrane and initiate the penetration process. We speculate that P22 VLPs are delivered via tail vein injection in a mouse model and may enter the liver and kidneys through the blood circulation and accumulate locally but fail to enter liver or kidney cells. Therefore, the ability of P22 VLPs-mCherry to penetrate primary cells needs to be further confirmed. Our results demonstrate that CPPs can significantly increase the efficiency with which P22 VLPs cross cell membranes and significantly improve the tissue distribution and retention of P22 VLPs in the lungs, brain, and spleen *in vivo*. The spleen is an important peripheral lymphoid organ containing abundant immune cells, such as antigen-presenting cells and T cells, and direct immunization of the spleen may induce a strong immune response. Wu et al. (48) reported that the accumulation of DNA vaccine-coated polymer nanoparticles in the spleen may trigger a burst of specific T-cell immunity. Therefore, our results suggest that TAT-conjugated P22 VLPs may directly deliver antigens to the spleen, thereby potentially enhancing the vaccine-induced immune response.

Kumar et al. (49) quantitatively characterized the biodistribution of various nanoparticles in different tissues after intravenous injection via nanoparticle biodistribution coefficients. Most nanoparticles accumulate in the liver and spleen but are less distributed in the lungs and brain. Organs such as the lung, heart, brain, and muscle contain a continuous endothelium, which forms a barrier for limiting nanoparticle delivery (50). We found that P22 VLP-mCherry is mainly distributed in the liver and kidneys and that CPP-conjugated P22 VLPs significantly promote the accumulation of VLPs in the lungs and brain. In addition, compared with the artificially synthesized polyarginine transmembrane peptide 8R, P22 VLPs conjugated with the TAT peptide derived from the HIV TAT protein transduction domain displayed greater delivery efficiency and superior biocompatibility in the liver, kidney, lung, and brain of mice. In addition, the size and materials of nanoparticles affect their tissue distribution. Previous studies have shown that large molecular graphene oxide (GO) nanoparticles (>200 nm) may exist in the gaps in the lungs because of their inability to pass through the pulmonary capillaries (51). Therefore, large GO nanoparticles can be used for selective drug delivery to the lungs. However, larger nanoparticles or aggregates in the lungs may increase dose-limiting toxicity (52). Although rapid uptake of larger silica nanoparticles was observed in the lungs within the first 6 h, their rapid clearance in the lungs and blood also occurred (49). Therefore, VLP-mCherry-TAT/8R particles of medium size (100–200 nm) may offer promising prospects for the delivery of drugs that target the lungs.

Previous studies have demonstrated that TAT can bind to protein with a molecular weight ranging from 36 to 119 kDa or inorganic quantum dots, thereby enhancing BBB absorption (53–55). Anand et al. demonstrated that TAT-modified P22 VLP-ziconotide (MVIIA) vesicles were taken up by human microvascular endothelial cells via the endocytic pathway (26). In this study, we demonstrated that TAT-conjugated P22 VLPs can accumulated in brain in a mouse model, which is consistent with the findings of a previous study (26). Besides, guanidine-rich CPPs have a strong affinity for negatively charged plasma membrane groups, efficiently entering various cell types in an energy-independent endocytic manner (34, 56). However, no direct evidence has shown whether 8R can cross the BBB. We observed that, compared with TAT, 8R more efficiently mediates the delivery of P22 VLPs to the brain, but this phenomenon is due to accumulation and adhesion of 8R-conjugated P22 VLPs in blood vessels or their crossing of vascular endothelial cells need to be verified by further study. Although the high charge density of arginine residues is beneficial for BBB permeability, the BBB influx transport properties cannot be correlated with their ability to penetrate the cell (57). Therefore, to increase the ability of P22 VLPs to penetrate nerve cells, ligand modification of P22 VLPs to target nerve cells may still be necessary. To facilitate the observation the penetration and tissue distribution of P22 VLPs, mCherry was selected as the model cargo in this study. Because the molecular weight and characteristic of different cargo will significantly affect construction strategy of P22 VLPs, which may impact the subsequent delivery and function of cargo. Therefore, it is necessary to directly verify the tissue distribution and delivery efficiency of CPPs-mediated P22 VLPs for functional proteins in future study.

In summary, we analyzed the effects of CPPs on improving the transport efficiency, tissue distribution and *in vivo* retention of P22 VLPs. These results demonstrated that the introduction of TAT and 8R could significantly increase the cellular uptake efficiency of VLPs. In particular, in the *in vivo* experiments, TAT exhibited good tissue distribution and retention in the lungs, whereas 8R exhibited greater accumulation in brain. In addition, TAT-modified P22 VLPs were found to have a lower impact on cytotoxicity than 8R VLPs did, suggesting that TAT may be superior as a drug or vaccine delivery system. By providing an in-depth understanding of the biocompatibility and tissue retention of CPPs, this study will expand their potential applications in the tissue-targeted delivery of macromolecular cargoes such as biological drugs and subunit vaccines.

## Data Availability

The original contributions presented in the study are included in the article/Supplementary material, further inquiries can be directed to the corresponding authors.
