# Supplementary material for: Cell-penetrating peptides TAT and 8R functionalize P22 virus-like particles to enhance tissue distribution and retention in vivo
Source: Front Vet Sci. 2024 Sep 3;11:1460973. doi: 10.3389/fvets.2024.1460973 (PMC11405305; doi:10.3389/fvets.2024.1460973)
Supplement: Supplementary file 1 [file Image_1.pdf]

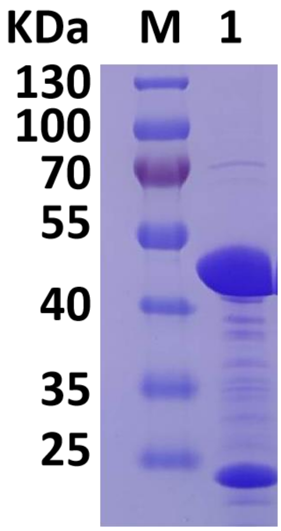

Figure S1. Characterization of the expression of P22 VLP by SDS−PAGE. M: protein marker; lines 1: with 0.1 mM IPTG induction of the indicated P22 VLP.

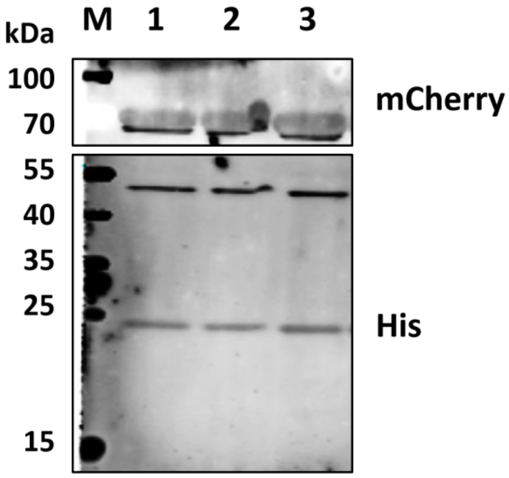

Figure S2 Identification of three mCherry-conjugated P22 VLPs components by Western blot (WB). M: protein marker; line 1-3: purified P22 VLP-mCherry, P22 VLP-mCherry-TAT, and P22 VLP-mCherry-8R. The 75 kDa band corresponded to the mCherry-modified coat protein (CP), while the unmodified CP and SP were observed at 50 kDa and 22.5 kDa, respectively.
