# Supplementary material for: Cell-penetrating peptides TAT and 8R functionalize P22 virus-like particles to enhance tissue distribution and retention in vivo
Source: Front Vet Sci. 2024 Sep 3;11:1460973. doi: 10.3389/fvets.2024.1460973 (PMC11405305; doi:10.3389/fvets.2024.1460973)
Supplement: Supplementary file 2 [file Table_1.docx]

**TABLE 1** Primers used for construction of plasmid pET-28a-P22VLP-mCherry, pET-28a-P22VLP-mCherry-TAT and pET-28a-P22VLP-mCherry-8R

| **Primer name** | **Sequence (5’-3’)** | **Purpose** |
| --- | --- | --- |
| mCherry-F | GACTGCGGGGTGGAGGCGGATCCGTGAGCAAGGG  CGAGGAGGA | pET-28a-P22VLP -mCherry |
| mCherry-R | TGTTAGCAGCCGGATCTTACTTGTACAGCTCGTCCA |  |
| P22-R | GGATCCGCCTCCACCCGCAGTCTGACCAGGCAGGC |  |
| P22-F | GACGAGCTGTACAAGGATCCGGCTGCTAACAAAGC |  |
| TAT-F | GAAAGAAGAGAAGACAAAGAAGAAGATAAGATCC  GGCTGCTAACAA | pET-28a-P22VLP  -mCherry-TAT |
| TAT-R | CTTCTTTGTCTTCTCTTCTTTCTACCGTACTTGTACAG  CTCGTCCATGC |  |
| 8R-F | GACGAGCTGTACAAGCGCCGACGCCGTCGCCGACG  CCGTTAAGATCCGGCTGCTAACA | pET-28a-P22VLP  -mCherry-8R |
| 8R-R | AGCAGCCGGATCTTAACGGCGTCGGCGACGGCGTC  GGCGCTTGTACAGCTCGTCCATGCCG |  |
